# Supplementary material for: HIVprotI: an integrated web based platform for prediction and design of HIV proteins inhibitors
Source: J Cheminform. 2018 Mar 9;10:12. doi: 10.1186/s13321-018-0266-y (PMC5845081; doi:10.1186/s13321-018-0266-y)
Supplement: Supplementary file 2 — Additional file 2. Source code of HIVProtI web server. [file 13321_2018_266_MOESM2_ESM.zip › HIVprotI_Source-code/hivprotI/weka-3-6-6/doc/weka/classifiers/lazy/kstar/KStarConstants.html]

KStarConstants


---


|  |  |  |
| --- | --- | --- |
| **Overview** \| **Package** \| **Class** \| **Tree** \| **Deprecated** \| **Index** \| **Help** \| **Weka's home** | |  |
| **PREV CLASS**   **NEXT CLASS** | **FRAMES**    **NO FRAMES**     **All Classes** |  |
| SUMMARY: NESTED \| FIELD \| CONSTR \| METHOD | DETAIL: FIELD \| CONSTR \| METHOD |  |


---


## weka.classifiers.lazy.kstar Interface KStarConstants

**All Known Implementing Classes:**: KStar, KStarNominalAttribute, KStarNumericAttribute

---

``` public interface KStarConstants ```

---

| **Field Summary** | |
| --- | --- |
| `static int` | `B_ENTROPY` |
| `static int` | `B_SPHERE`             Blend setting modes |
| `static double` | `EPSILON` |
| `static double` | `FLOOR` |
| `static double` | `FLOOR1` |
| `static double` | `INITIAL_STEP` |
| `static double` | `LOG2` |
| `static int` | `M_AVERAGE` |
| `static int` | `M_DELETE`             Missing value handling mode |
| `static int` | `M_MAXDIFF` |
| `static int` | `M_NORMAL` |
| `static int` | `NUM_RAND_COLS` |
| `static int` | `OFF` |
| `static int` | `ON`             Some usefull constants |
| `static double` | `ROOT_FINDER_ACCURACY` |
| `static int` | `ROOT_FINDER_MAX_ITER`             How close the root finder for numeric and nominal have to get |

**Field Detail**

### ON

```
static final int ON
```

:   Some usefull constants

    **See Also:**: Constant Field Values

---


### OFF

```
static final int OFF
```

**See Also:**: Constant Field Values

---


### NUM\_RAND\_COLS

```
static final int NUM_RAND_COLS
```

**See Also:**: Constant Field Values

---


### FLOOR

```
static final double FLOOR
```

**See Also:**: Constant Field Values

---


### FLOOR1

```
static final double FLOOR1
```

**See Also:**: Constant Field Values

---


### INITIAL\_STEP

```
static final double INITIAL_STEP
```

**See Also:**: Constant Field Values

---


### LOG2

```
static final double LOG2
```

**See Also:**: Constant Field Values

---


### EPSILON

```
static final double EPSILON
```

**See Also:**: Constant Field Values

---


### ROOT\_FINDER\_MAX\_ITER

```
static final int ROOT_FINDER_MAX_ITER
```

:   How close the root finder for numeric and nominal have to get

    **See Also:**: Constant Field Values

---


### ROOT\_FINDER\_ACCURACY

```
static final double ROOT_FINDER_ACCURACY
```

**See Also:**: Constant Field Values

---


### B\_SPHERE

```
static final int B_SPHERE
```

:   Blend setting modes

    **See Also:**: Constant Field Values

---


### B\_ENTROPY

```
static final int B_ENTROPY
```

**See Also:**: Constant Field Values

---


### M\_DELETE

```
static final int M_DELETE
```

:   Missing value handling mode

    **See Also:**: Constant Field Values

---


### M\_MAXDIFF

```
static final int M_MAXDIFF
```

**See Also:**: Constant Field Values

---


### M\_NORMAL

```
static final int M_NORMAL
```

**See Also:**: Constant Field Values

---


### M\_AVERAGE

```
static final int M_AVERAGE
```

**See Also:**: Constant Field Values


---


|  |  |  |
| --- | --- | --- |
| **Overview** \| **Package** \| **Class** \| **Tree** \| **Deprecated** \| **Index** \| **Help** \| **Weka's home** | |  |
| **PREV CLASS**   **NEXT CLASS** | **FRAMES**    **NO FRAMES**     **All Classes** |  |
| SUMMARY: NESTED \| FIELD \| CONSTR \| METHOD | DETAIL: FIELD \| CONSTR \| METHOD |  |


---
